# Oriented Polyaniline Nanowire Arrays Grown on Dendrimer (PAMAM) Functionalized Multiwalled Carbon Nanotubes as Supercapacitor Electrode Materials

**DOI:** 10.1038/s41598-018-24265-7

**Published:** 2018-04-19

**Authors:** Lin Jin, Yu Jiang, Mengjie Zhang, Honglong Li, Linghan Xiao, Ming Li, Yuhui Ao

**Affiliations:** College of Chemistry and Life Science, Jilin Province Key Laboratory of Carbon Fiber Development and Application, Changchun University of Technology, Changchun, 130012 People’s Republic of China

## Abstract

At present, PANI/MWNT composites have been paid more attention as promising electrode materials in supercapacitors. Yet some shortcomings still limit the widely application of PANI/MWNT electrolytes. In this work, in order to improve capacitance ability and long-term stability of electrode, a multi-amino dendrimer (PAMAM) had been covalently linked onto multi-walled carbon nanotubes (MWNT) as a bridge to facilitating covalent graft of polyaniline (PANI), affording P-MWNT/PANI electrode composites for supercapacitor. Surprisingly, ordered arrays of PANI nanowires on MWNT (setaria-like morphology) had been observed by scanning electron microscopy (SEM). Electrochemical properties of P-MWNT/PANI electrode had been characterized by cyclic voltammetry (CV) and galvanostatic charge-discharge technique. The specific capacitance and long cycle life of P-MWNT-PANI electrode material were both much higher than MWNT/PANI. These interesting results indicate that multi-amino dendrimer, PAMAM, covalently linked on MWNT provides more reaction sites for *in-situ* polymerization of ordered PANI, which could efficiently shorten the ion diffusion length in electrolytes and lead to making fully use of conducting materials.

## Introduction

Supercapacitors are considered as promising high-power sources due to their high power performance, long cycle life and low cost^1,2^. PANI/carbon-based composites had been intensively investigated as a promising candidate for supercapacitors not only due to their low cost and high conductivity but also the synergistic performances of large pseudocapacitance^3–8^.

Capacitance and cycling stability were two of critical factors of the hybrid electrode for supercapacitors. Conducting polymer, including PANI, tends to distort in the long term charge-discharge process and then led to peeling off from the carbon materials^9–11^, consequently deteriorating the performance of composites^11^. Apart from cycling stability, physical contact between carbon materials and PANI may also prevent the charge transfer which may decrease the overall capacitance of composites^12^. In addition, the morphology and distribution of polymers which facilitate fast transport of electrons and ions are difficult to control precisely^13,14^. For these sake, Covalent connection had been considered as an efficient method to fix the high capacitive polymers onto the carbon materials^15–17^. As Kumar reported^18^, they prepared highly conducting polyaniline-grafted reduced graphene oxide (PANI-g-rGO) composites by functionalizing graphite oxide with 4-aminophenol via acyl chemistry. PANI could be *in-situ* polymerized on the amino-functionalized r-GO, affording PANI-g-rGO with larger capacitance (250 F/g) than PANI-rGO. To fully use the inner layer of conductive polymers and maximize the capacitance, several methods for tuning morphology of PANI had been developed^19–21^. Oriented arrays especially vertical arrays of PANI nanowires were one of the attractive nanostructures due to efficiently direct electronic pathways for charge transport which consequently bring high capacitance^21–23^. Incorporation of the multi amino group, octa (amino-phenyl) silsesquioxane(OASQ), for assisting the formation of the hyper branched PANI provide a promising alternative method for fabricating 3D micro-structure of conducting polymers^12^. However, to the best of our knowledge, the morphology of conducting polymer grafting on carbon materials covalently, especially the PANI grown on carbon nanotubes for supercapacitors hasn’t been well investigated.

**Figure 1:**
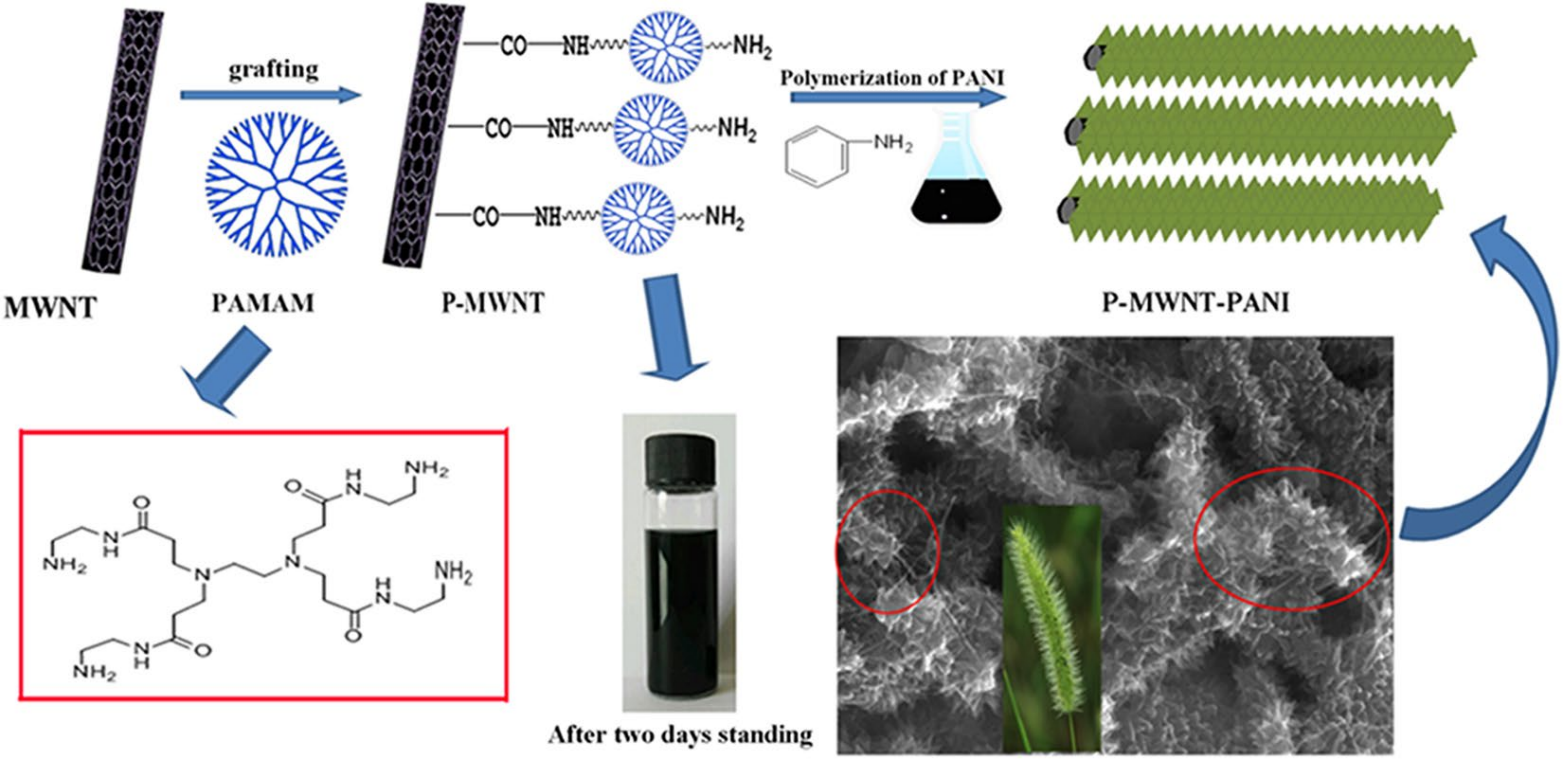
Schematic representation for preparation of P-MWNT-PANI.

In this work, multi amino dendrimer PAMAM was introduced onto CNTs for assisting *in situ* polymerization of aniline grown on multiwalled carbon nanotubes (MWNTs) orderly, affording P-MWNT/PANI hybrid electrodes. Carbon nanotubes (CNTs) had been recognized as one of an attractive one-dimensional electrode materials for energy storage devices, which is attributable to the excellent chemical and electrochemical stability, highly accessible surface area and excellent electrical conductivity^24,25^. Dendrimer PAMAM has higher amine density which improves the reaction possibility, resulting in a higher grafting density. In addition, PAMAM can easily form a uniform film on a substrate by self-assembly ensuring the uniformity of MWNT^26,27^. Comparing with directly functionalized MWNT with –NH_2_, higher amine density on the CNTs originated from PAMAM might prevent the aggregation of carbon materials during *in-situ* polymerization of aniline^17,18^ and result in a higher grafting density of PANI. PAMAM covalently linked PANI and MWNT might provide more bridges for facilitating charge transfer. More importantly, the dendrimer structure with more active sites on the CNTs probably induced the unique micro-structure of the PANI/MWNT hybrid electrode. Therefore, incorporation of covalently linked dendrimer PAMAM onto the CNTs is not only to increase the cycling stability and conductivity of the hybrid electrode but also to increase the electrode/electrolyte contact surface area for fully using the inner layer of conducting polymer. The fabrication of P-MWNT-PANI was illustrated in Fig. 1. Compared with PANI growing on pristine MWNT by Van der Waals attraction, the excellent specific capacitance as high as 568 F g^−1^ was observed and good cyclic stability was also exhibited in this electrode. While for PANI and MWNT/PANI the capacitance were 180 F g^−1^ and 250 F g^−1^, respectively. These results demonstrate that the synergistic effects between PANI and functionalized MWNT significantly affect the electrochemical performance of supercapacitor electrodes.

## Results and Discussion

### Morphological and structural characterization

**Figure 2:**
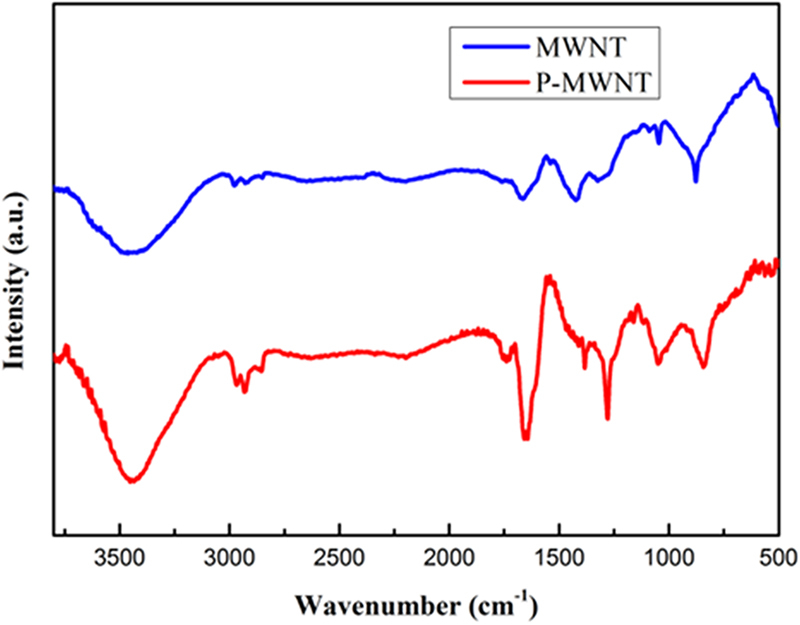
FT-IR spectra of MWNT and P-MWNT.

**Figure 3:**
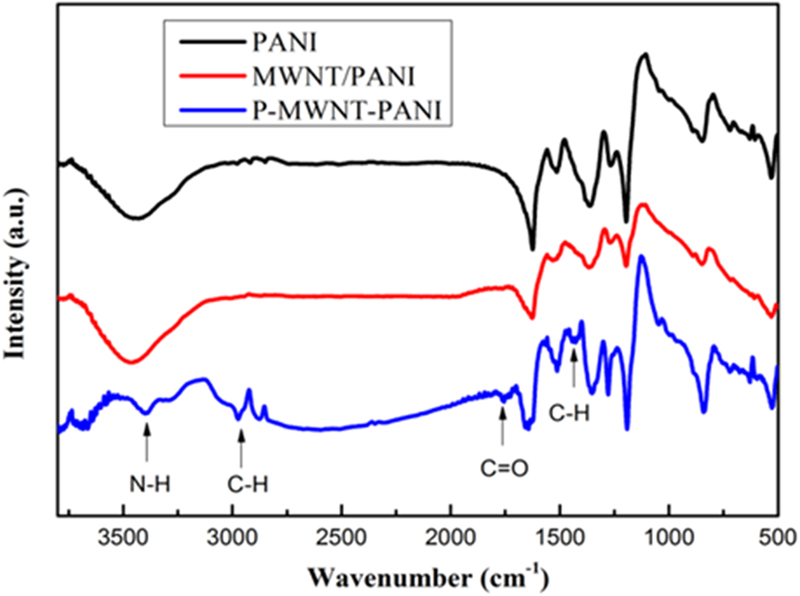
FT-IR spectra of PANI, MWNT/PANI, P-MWNT-PANI.

FT-IR measurement was carried out to characterize the chemical structure of materials. As shown in Fig. 2, the peak located at 1280 cm^−1^ and 1660 cm^−1^ were attributed to the absorbance of C-N and C=O bands of amide group clearly demonstrating the successful functionalization of MWNT by PAMAM^28^. As shown in Fig. 3, all the characteristic bands of PANI chains can be observed. The bands at 1580 cm^−1^ and 1515 cm^−1^ are assigned to the stretching vibration of C=C in the quinonoid and benzenoid rings, respectively^29^. The peak at 1276 cm^−1^ is the stretching vibrations of the secondary amide group. Though some similar absorption bands were observed in the spectra of composites, but with a red shift of P-MWNT-PANI (PANI: 1515 cm^−1^; MWNT/PANI: 1515 cm^−1^; P-MWNT-PANI: 1505 cm^−1^), indicating chemical interactions existed between functionalized MWNT and PANI. In addition, the stretching vibrational band of C=O of the imide group appears in spectra also verifying covalent connections in composites^12^.

**Figure 4:**
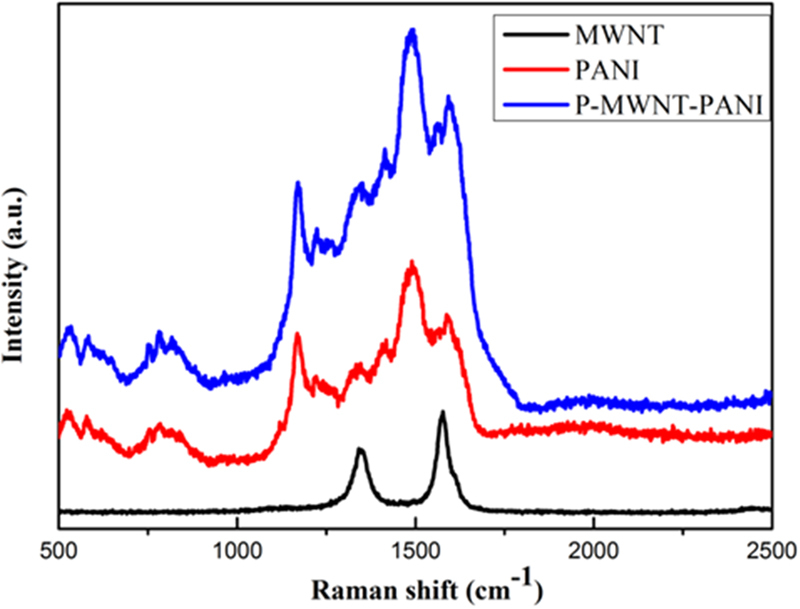
Raman spectra of MWNT, PANI and P-MWNT-PANI.

The interaction between P-MWNT and PANI nanofibers was further investigated using Raman spectrum analysis. As shown in Fig. 4, the Raman spectrum of MWNT displays two prominent peaks at 1340 and 1580 cm^−1^, corresponding to the D-band (C-C, disordered graphite structure) and G-band (sp^2^ hybridized carbon), respectively^30^. For pure PANI and P-MWNT-PANI, the band at 1169, 1223, 1338, 1487 and 1588 cm^−1^ are assigned to in-plane C-H bending of quninoid ring, in-plane C-H bending of the benzenoid ring, the C-N•^+^ stretching vibration, C=C stretching of the quinoid ring, and C=C stretching of the benzenoid ring, indicating the presence of a doped PANI structure^30^. Notably, comparing with the PANI there is a blue shift in the position of the G peak (from 1588 to 1595 cm^−1^) for P-MWNT-PANI, indicating the charge carrier concentration is significantly altered due to the strong interaction between P-MWNT and PANI^31^. On the basis of above results, these functional groups can act as anchor sites and enable *in situ* formation of nanostructure.

**Figure 5:**
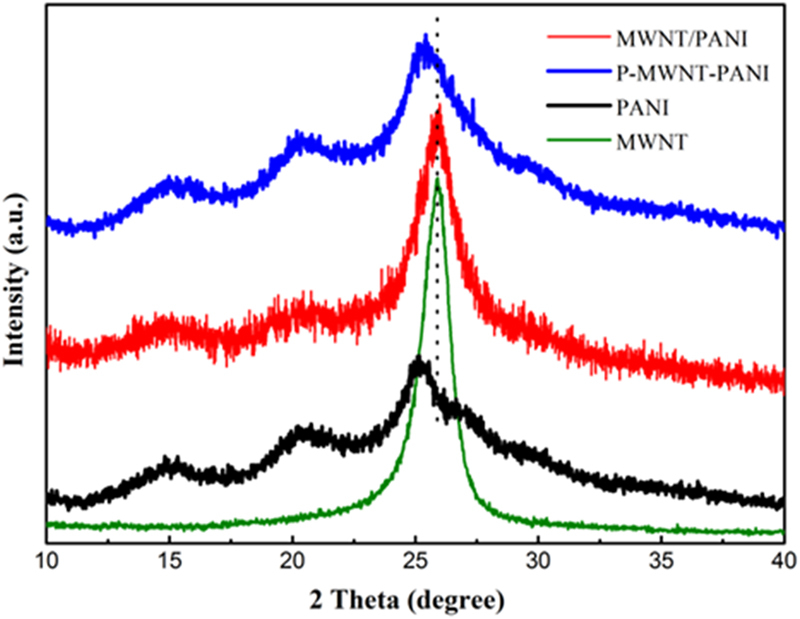
XRD pattern of PANI, MWNT, MWNT/PANI and P-MWNT-PANI.

Moreover, the XRD investigation result confirms the crystal form transformation of PANI nanofibers on MWNT. Figure 5 presents the X-ray diffraction data for MWNT, MWNT/PANI and P-MWNT-PANI. The MWNT shows an intense and sharp peak at 2θ = 25.9°. The peaks appear at 2θ = 14.8°, 20.7° and 25.3°, corresponding to (011), (020) and (200) crystal planes of PANI^32,33^. For MWNT/PANI composites, the crystalline peaks are similar to those obtained from the pure PANI, implying that the composites have not acquired additional crystalline structure^34^. However, the diffraction peak at 2θ = 25.9° were also observed, indicating that the MWNT have not fully interacted with PANI^17^. It is worthy that the peak of MWNT has almost disappeared in P-MWNT-PANI according to the data. The result suggests that PANI has been uniformly covered onto the P-MWNT substrates^35,36^.

**Figure 6:**
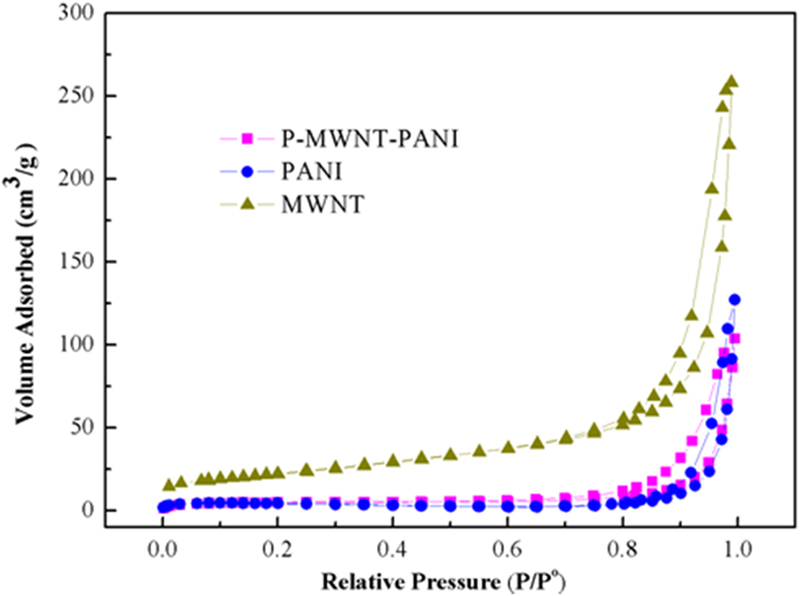
N_2_ adsorption and desorption isotherms of PANI, P-MWNT-PANI and MWNT.

Figure 6 shows the N_2_ absorption-desorption isotherms for PANI and P-MWNT-PANI nanocomposites. As can be seen from Fig. 6 the N_2_ absorption-desorption isotherms of samples exhibit type IV sorption behavior, which represents the mesoporous structure characteristics. The BET specific surface area of PANI is estimated to be 10.88 m^2^ g^−1^. While compared with PANI, the value of P-MWNT-PANI is 15.53 m^2^ g^−1^, higher than pristine PANI. It could be considered that successful growing ordered PANI nanowires on functionalization MWNT actually improve the BET specific surface area. This improvement may provide a large number of active sites for charge-transfer reactions. The value of MWNT is 80.22 m^2^ g^−1^. Compared with P-MWNT-PANI composites, this remarkable decrease of specific surface area is attributed to the increase of more compact morphology, larger diameter for P-MWNT-PANI and disappearance of micropore after growing ordered PANI nanofibers^37–39^.

**Figure 7:**
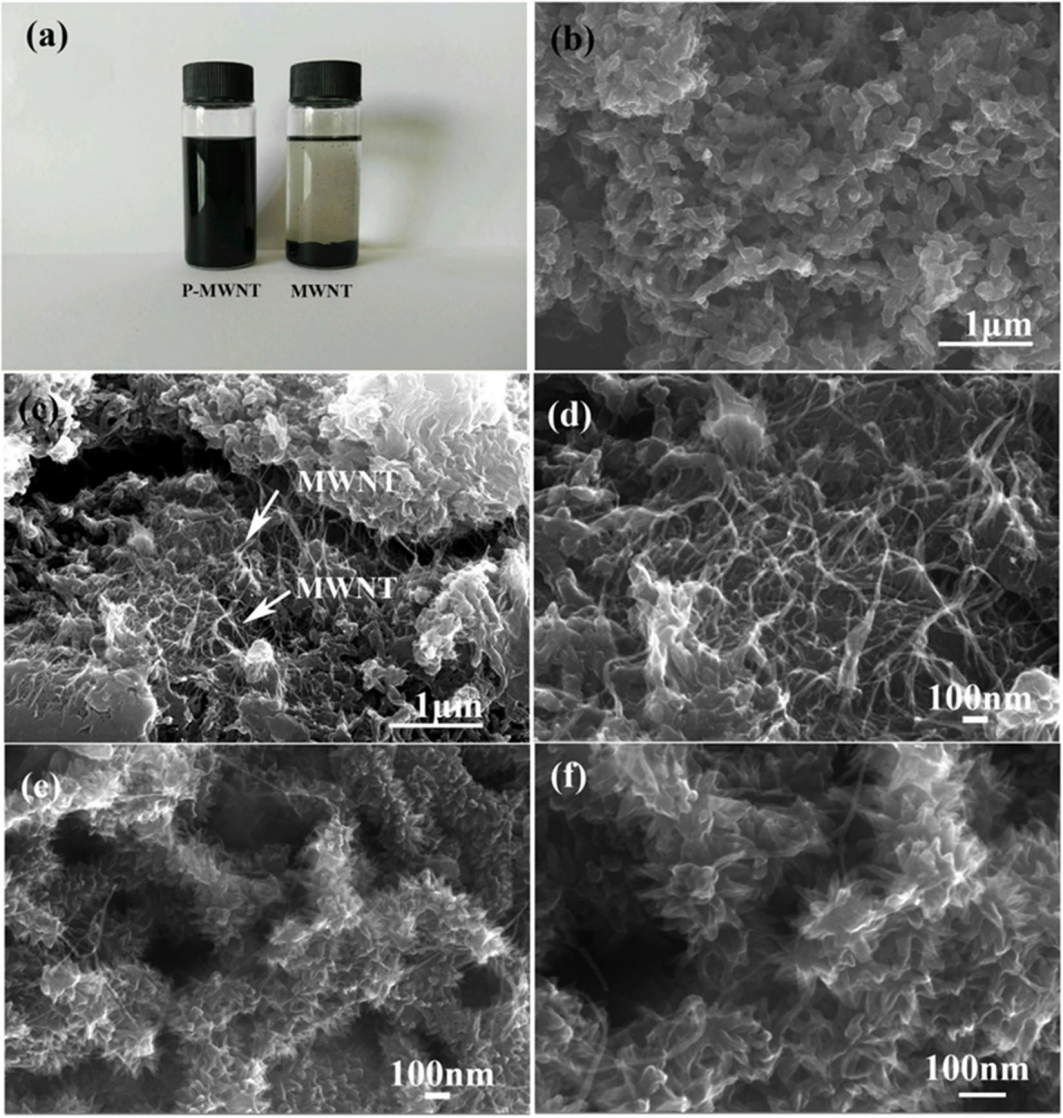
(**a**) Photograph of alcohol solutions containing of MWNT and P-MWNT. (**b**) SEM image of pure PANI. (**c**) and (**d**) SEM images of MWNT/PANI at different magnifications. (**e**) and (**f**) SEM images of P-MWNT-PANI at different magnifications.

Figure 7(a) is the photograph of vials containing alcohol dispersion of MWNT and P-MWNT after two days standing. As shown in picture, the MWNT suspension coagulated, whereas homogenous P-MWNT suspension has been obtained after functionalization. TEM micrographs of the MWNT and P-MWNT are given in Figure S1. The MWNT presents entangled agglomerates randomly as a result of their extremely strong hydrophobic nature. While after grafting PAMAM, less agglomeration of MWNT was observed. These results indicate that grafting PAMAM on MWNT was beneficial for dispersion effectively. The morphology of composites was investigated by SEM. The pristine PANI exhibits randomly nanofiber structure shown in Fig. 7(b). In MWNT/PANI composites as shown in Fig. 7(c,d), disordered nanofiber similar phenomenon is observed, besides, agglomeration of MWNT occurs. For MWNT/PANI composites, there was no covalent connection between MWNT and PANI only weak Van der Waals interactions existed as a result of poor guiding role in PANI growth. However, different from their morphology, the SEM images in Fig. 7(e,f) indicate that the as-prepared P-MWNT-PANI composites form uniform and acanthine PANI nanowires deposited onto MWNT substrates. The variation in the PANI morphology of composites confirmed our assumption that the amino groups on the MWNT initiated the polymerization of aniline, which guided the growth of PANI, thus formed an ordered structure. On the basis of the PANI deposition, the highly ordered nanostructure may lead to the fast transport of electrons and ions in the supercapacitor devices.

### Electrochemical properties

**Figure 8:**
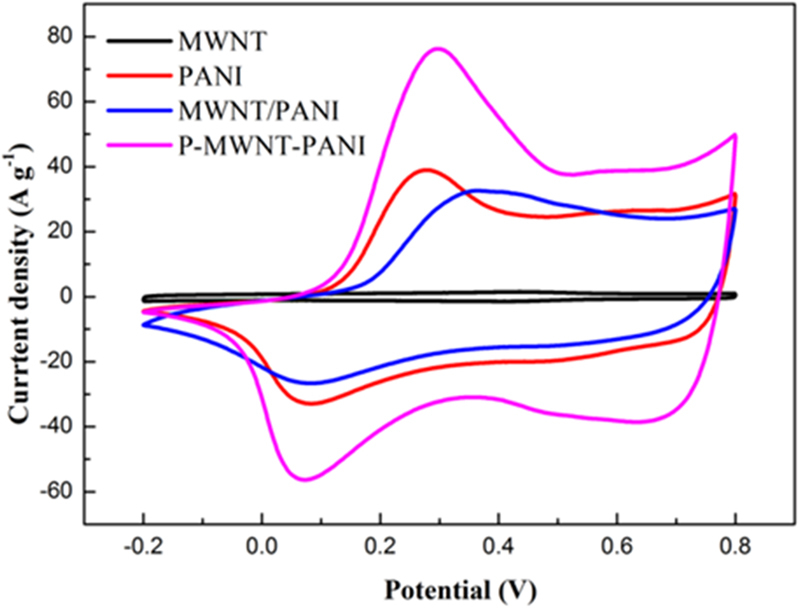
Cyclic voltammograms (CV) curves of the MWNT, PANI, MWNT/PANI and P-MWNT-PANI electrodes at scan rate of 100 mV s^−1^.

**Figure 9:**
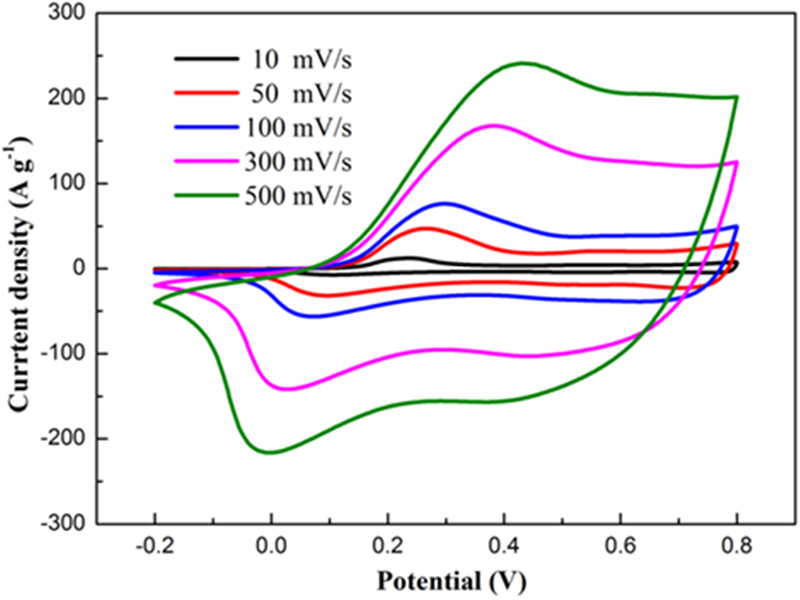
CV curves of P-MWNT-PANI electrode at different scan rates.

Electrochemical tests were carried out using cyclic voltammetry (CV) and galvanostatic charge-discharge techniques to measure the electrochemical performance of the composites. Figure 8 shows the CV curves of different electrodes within potential range of −0.2–0.8 V at a scanning rate of 100 mV s^−1^. Pairs of redox peaks of PANI, MWNT/PANI and P-MWNT-PANI are found on the CV curves owing to the redox converting procedures of PANI. The curve of MWNT has no redox peak indicating the typical EDL energy storage mechanism^28^. The CV curve of MWNT/PANI is featureless compared with PANI. Notably, the area and the current density of the P-MWNT-PANI were much higher than those of bare PANI and MWNT/PANI at the same sweeping rate, indicating a greatly enhanced specific capacitance. The improvement of performance can be attributed to the combined effect of chemical bonding and the unique microstructure of PANI. The CV curves of P-MWNT-PANI at different sweeping rates also obtained in Fig. 9. The features of the P-MWNT-PANI electrode curve at high sweeping rates are similar to those at low scan rates, the redox peaks enhance apparently with the sweeping rate increasing, indicating a good rate capability for P-MWNT-PANI composite^40,41^.

**Figure 10:**
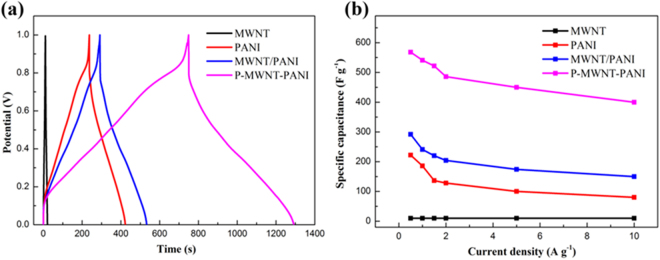
(**a**) Charge-discharge curves of MWNT, PANI, MWNT/PANI and P-MWNT-PANI nanocomposites at a current density of 1 A g^−1^. (**b**) Specific capacitance of MWNT, PANI, MWNT-PANI and P-MWNT at different current densities.

Figure 10(a) shows the galvanostatic charge-discharge curves of samples at a current of 1 A g^−1^. The gravimetric specific capacitance results of various composites were calculated from the discharge curves shown in Fig. 10(b). Pure MWNT electrode exhibits triangular charge-discharge branches, suggesting the typical EDL capacitance with rapid charge-discharging process. P-MWNT-PANI shows a longer discharge time compared with that of PANI and MWNT/PANI. The specific capacitance of pure PANI is estimated to be 180 F g^−1^ at the current density of 1 A g^−1^. While for MWNT/PANI, the specific capacitance is enhanced to 250 F g^−1^ higher than pure PANI material which may attribute to the incorporation of highly conductive MWNT, facilitating a rapid transport of electrolyte ions within the electrode material. The capacitance is about 540 F g^−1^ obtained in P-MWNT-PANI at the same current density. This significant improvement related to our composites include: (1) P-MWNT substrate serve as a 3D conductive skeleton that direct path for electrons within the composites; (2) the P-MWNT network act as a template for growth of ordered PANI, which provides larger contact surface area and shortens the path length for electrolyte ion transport; (3) grafting MWNT on PANI using covalent bonding enhance the interactions as a result of reducing the interfacial resistance of each components. The gravimetric specific capacitance results of various composites with different current densities were calculated from the discharge curves shown in Fig. 10(b). Figure S2 displays all the galvanostatic charge-discharge curves of P-MWNT-PANI at different rates. Notably, at all current densities (0.5–10 A g^−1^), the capacitance of P-MWNT-PANI is higher than PANI and MWNT/PANI. The highest value for P-MWNT-PANI achieved is 568 F g^−1^ at 0.5 A g^−1^. The capacitance of P-MWNT-PANI has 400 F g^−1^ even at 10 A g^−1^ (70% retention of the capacitance at 0.5 A g^−1^), making it very promising for rapid charge-discharge applications. The improved rate capability of P-MWNT-PANI composites may be due to the incorporation of the P-MWNT as a mechanical support not only guide ordered nanostructure of growing PANI, but also shorten the ion diffusion length of the electrolyte and active materials.

**Figure 11:**
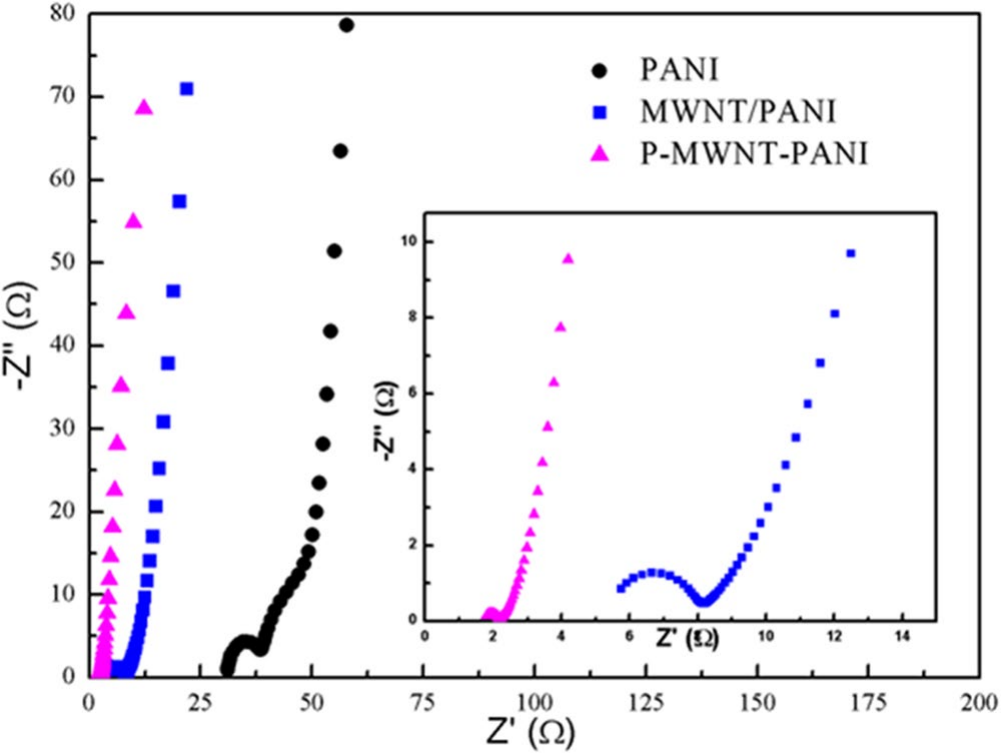
Nyquist plots of PANI, MWNT/PANI and P-MWNT-PANI at a frequency range from 100 kHz to 0.01 Hz.

The electrochemical impedance spectroscopy (EIS) analysis is one of the important methods to examine impedance of electrode composites. The Nyquist plot was shown in Fig. 11 with an inset of high frequency region. The equivalent circuit is given in the Figure S3, where *R*_s_ is the solution resistance obtained from the interception of the Nyquist plots at the X axis, *R*_ct_ is corresponding to the charge transfer resistance which can be measured as the diameter of the semicircle, *Z*_w_ represents the Warburg resistance^10,35^. At low frequency, MWNT/PANI and P-MWNT-PANI composites exhibit a more vertical line than pure PANI, indicating a better capacitor behavior^42^. As shown in the data, pure PANI shows the highest electrolyte solution resistance (30.96 Ω). This implies that random PANI nanofibers are not favor of the electrolyte ion diffusion to the ploymer surface on the one hand, on the other hand, the aggregations of PANI nanofibers hinder the using of the inner layer active materials. However, the *R*_s_ of MWNT/PANI and P-MWNT-PANI are 5.41 Ω and 1.73 Ω, much less than pure PANI, indicating that the incorporation of MWNT can facilitate ion diffusion. Besides, it can be clearly seen that *R*_ct_ of P-MWNT-PANI composite (0.40 Ω) is smaller than PANI (21.89 Ω) and MWNT/PANI (2.45 Ω), which demonstrates that this oriented PANI structure provides larger contact surface area and is beneficial to the efficient access of electrolyte ion to the surface PANI. The above data is in accordance with results of CV curves and galvanostatic charge-discharge curves.

**Figure 12:**
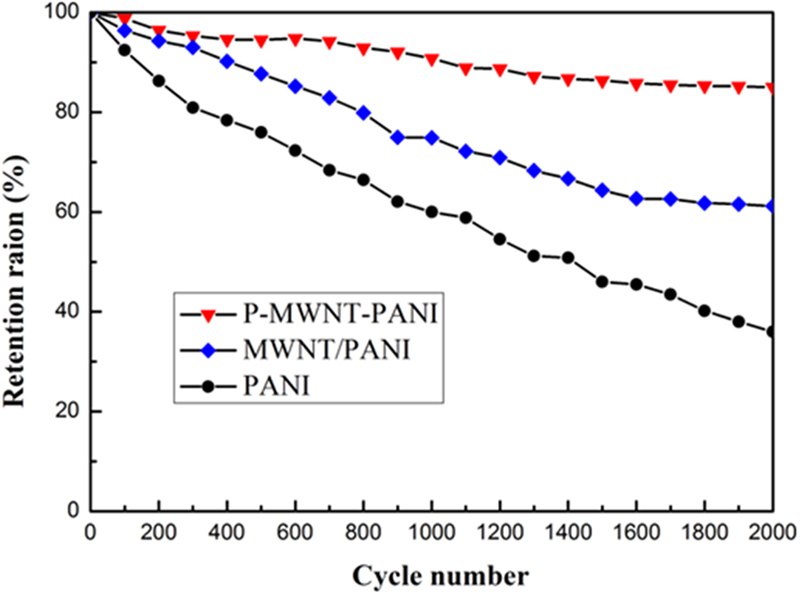
Cycling performance of PANI, MWNT/PANI and P-MWNT-PANI composite at a current density of 2 A g^−1^.

Cycling stability of capacitor is a crucial factor determining its long term application. Figure 12 shows the capacitance retention of composites at a constant current density of 2 A g^−1^ with 2000 charge-discharge cycles. Under the successive charging-discharging cycle, the specific capacitance of P-MWNT-PANI composites retain 85.0% retention of the initial capacitance further indicating both high rate performance and excellent long-term stability. The capacitance of MWNT/PANI retains almost 61.2% of its initial value due to the high cycling stability of MWNT. The capacitance of sole PANI continues to decrease throughout the cycling process, the value decreased from 225 to 80 F g^−1^, which is about 35.6% of its initial value indicating poor stability. These results indicate that the connection between the active material and electrolyte is improved via covalent bonding, and full use is made of PANI in the electrode, suggesting a highly promising prospective for electrode materials.

## Conclusions

In summary, a hybrid electrode material of polyaniline (PANI) nanofiber and multiwalled carbon nanotubes (MWNT) was prepared by introducing a poly (amido amine) (PAMAM) dendrimer as chemically “bridge” between MWNT and PANI. The polyaniline, growing at the anchor sites of functionalized MWNT, exhibits the ordered nanorods structure as SEM images exhibited. According to the electrochemical test, the composites showed excellent specific capacitances and cycling stability than pristine PANI and MWNT/PANI as supercapacitor electrodes. Based on the experimental results, we believed that the synergistic effect between the two components leading to remarkable improvements of composites. This study supplies an effective way to obtain a promising candidate for appealing technological application in energy storage systems.

## Materials and Methods

### Materials

Concentrated sulfuric acid (H_2_SO_4_, 98%), nitric acid (HNO_3_, 68%), hydrochloric acid (HCl), alcohol, N,N-dimethylformamide (DMF), acetone, ammonium persulfate (APS) and aniline were purchased from Aladdin Co, Shanghai China. Aniline was distilled under reduced pressure and stored in dark room prior to use. The poly-(amidoamine) (PAMAM) dendrimers (Generation 0) with ethylenediamine core was provided by Shanghai Chemical Co. Multiwalled carbon nanotubes(MWNT), with diameter of ~20 nm, was obtained from Hengqiu Co, Suzhou China.

### Preparation of P-MWNT

The MWNT were treated in a 3:1 (by volume) mixture of concentrated nitric and sulfuric acid for 2 h at 80 °C to introduce carboxylic group, then washed with deionized water until the pH value was 7, followed by drying in vacuum for 24 h. Next, 300 mg COOH-MWNT was reacted with 200 ml SOCl_2_ and 5 ml DMF at 80 °C for 48 h with nitrogen protection, and the vent was connected to a CaCl_2_ guard tube to protect from atmosphere water. After the acyl chlorination reaction, the excess SOCl_2_ was removed by distillation under reduced pressure at 90 °C. The product named COCl-MWNT was washed with acetone for several times. Finally, the obtained powders were reacted with 1 × 10^-4^ mol PAMAM under nitrogen atmosphere for 12 h. After that, the mixture was collected by suction filtration and washed with acetone and deionized water then dried at 60 °C for 12 h, leading to P-MWNT.

### Synthesis of P-MWNT-PANI

Polyaniline-grated P-MWNT (P-MWNT-PANI) was prepared via an *in situ* polymerization in the presence of P-MWNT. In short, 50 mg P-MWNT was added to 100 ml of 1 M HCl aqueous solution by ultrasonication for 1 h at room temperature, then 50 ml 1 M HCl aqueous solution containing 1.49 g aniline was added into the above solution. Another 1.824 g APS containing 50 ml of 1 M HCl aqueous solution was rapidly added then stirred for 30 s. After reacting for 24 h in ice-water bath, the obtained product was filtrated with 0.22 μm porous membrane. The filtrate cake was dried in a vacuum dry oven at 45 °C.

For comparison, the PANI nanofiber was generated in the same way without P-MWNT. For synthesis of MWNT/PANI, 50 mg MWNT was used and the rest of procedures were the same as synthesis of PANI.

### Characterization

The Fourier transform infrared (FTIR) was used to determine the chemical structure of the samples within the 400–4000 cm^−1^ region (Bruker-IFS 66 V/S). The Raman spectra were measured using a LabRam HR Evolution Raman Spectrometer (Jobin-Yvon Horiba Scientific), equipped with a air cooled frequency doubled Nd:Yag 532 nm laser for excitation (100 mW). The laser was focused through a 50 objective. Structures of products were characterized by X-ray diffraction (XRD) pattern, using Rigaku D/max 2550. The Brunauer-Emmett-Teller (BET) specific surface area was calculated by using the Barrett-Joyner-Halenda (BJH) model. The morphology and structure of products were characterized by scanning electron microscopy (SEM, SUPRA 40–32). Transmission electron microscopy (TEM) images of samples are recorded in a TEOL-2000EX microscope at 100Kv.

### Electrochemical performance

All electrochemical measures were conducted on an electrochemical station (CHI 6601, Shanghai Chenhua) in a three-electrode system using a platinum sheet as the counter electrode, a AgCl/Ag electrode as reference electrode and a glassy-carbon electrode as the working electrode coated with samples that were impregnated with 5% Nafion solution. The measurements were carried out in 1 M H_2_SO_4_ aqueous electrolyte at room temperature. Cyclic voltammetry (CV) was carried out in a potential range of −0.2–0.8 V at scan rates of 10, 50, 100, 300 and 500 mV s^−1^. Galvanostatic charge-discharge properties were measured in a voltage range of 0–1 V. The specific capacitance of the electrode materials can be calculated according to the discharge branch by the following equation

1 $$C=\frac{I{\rm{\Delta }}t}{m{\rm{\Delta }}V}$$

Where *C* is the specific capacitance, *I* is the charge-discharge current, *ΔV* is the voltage window, *m* is the mass of the active material on working electrode and *Δt* is the discharge time^31^. The electrochemicalimpedance spectroscopy (EIS) was measured in the frequency range of 0.01 Hz–100 k Hz at open circuit potential with an ac perturbation of 5 mV.

## Electronic supplementary material

Supplementary Information
