# Supplementary material for: Oriented Polyaniline Nanowire Arrays Grown on Dendrimer (PAMAM) Functionalized Multiwalled Carbon Nanotubes as Supercapacitor Electrode Materials
Source: Sci Rep. 2018 Apr 19;8:6268. doi: 10.1038/s41598-018-24265-7 (PMC5908880; doi:10.1038/s41598-018-24265-7)
Supplement: Supplementary file 1 — Supplementary Information [file 41598_2018_24265_MOESM1_ESM.pdf]

Supplementary Information

Oriented Polyaniline Nanowire Arrays Grown on Dendrimer (PAMAM) Functionalized Multiwalled Carbon Nanotubes as Supercapacitor Electrode Materials

Lin Jin[a], Yu Jiang[a], Mengjie Zhang[a], Honglong Li[a], Linghan Xiao*[a], Ming Li*[a], Yuhui Ao*[a]

[a] College of Chemistry and Life Science, Jilin Province Key Laboratory of Carbon Fiber Development and Application, Changchun University of Technology, Changchun 130012, People's Republic of China.

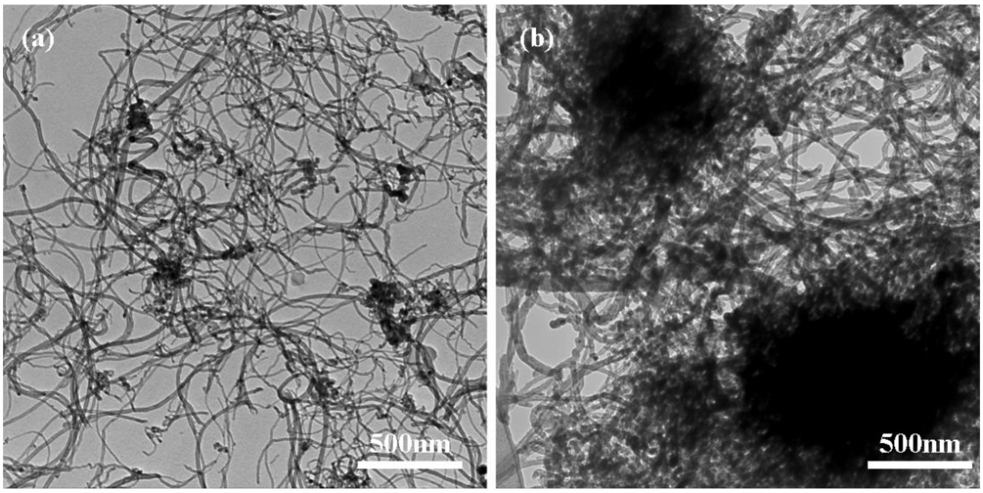

Figure S1.TEM micrographs of P-MWNT (a) and pristine MWNT (b).

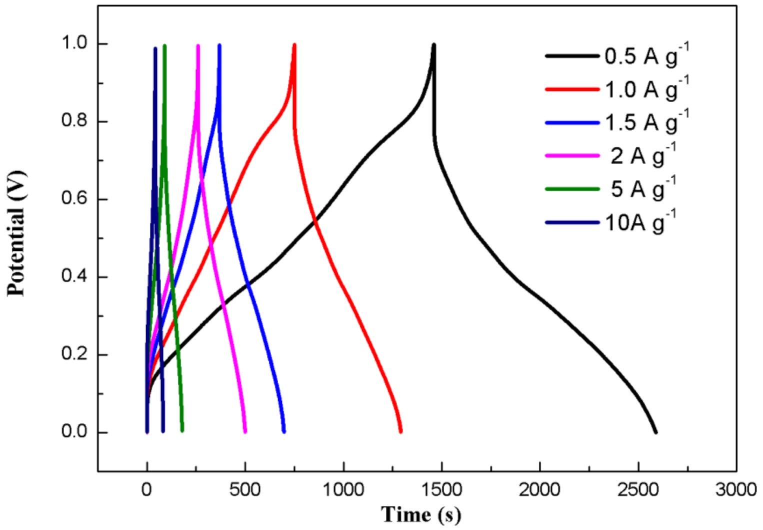

Figure S2. Charge-discharge curves with different rates of P-MWNT-PANI.

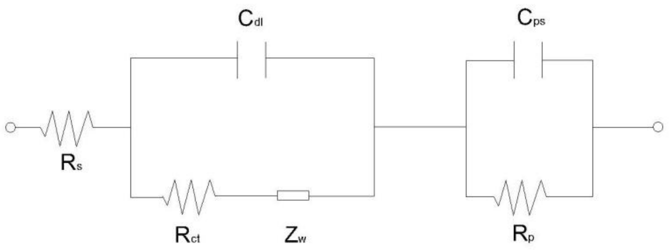

Figure S3. The equivalent electric circuit used in fitting the experimental data.
